# Adaptive Ant Colony Optimization with Sub-Population and Fuzzy Logic for 3D Laser Scanning Path Planning

**DOI:** 10.3390/s24041098

**Published:** 2024-02-08

**Authors:** Junfang Song, Yuanyuan Pu, Xiaoyu Xu

**Affiliations:** College of Information Engineering, Xizang Minzu University, No. 6, East Section of Wenhui Road, Weicheng District, Xianyang 712082, China; puyyuan@163.com (Y.P.); jingxu1993@163.com (X.X.)

**Keywords:** laser scan, intricate surface measurement, scan path planning, ant colony optimization, fuzzy logic

## Abstract

For the precise measurement of complex surfaces, determining the position, direction, and path of a laser sensor probe is crucial before obtaining exact measurements. Accurate surface measurement hinges on modifying the overtures of a laser sensor and planning the scan path of the point laser displacement sensor probe to optimize the alignment of its measurement velocity and accuracy. This manuscript proposes a 3D surface laser scanning path planning technique that utilizes adaptive ant colony optimization with sub-population and fuzzy logic (SFACO), which involves the consideration of the measurement point layout, probe attitude, and path planning. Firstly, this study is based on a four-coordinate measuring machine paired with a point laser displacement sensor probe. The laser scanning four-coordinate measuring instrument is used to establish a coordinate system, and the relationship between them is transformed. The readings of each axis of the object being measured under the normal measuring attitude are then reversed through the coordinate system transformation, thus resulting in the optimal measuring attitude. The nominal distance matrix, which demonstrates the significance of the optimal measuring attitude, is then created based on the readings of all the points to be measured. Subsequently, a fuzzy ACO algorithm that integrates multiple swarm adaptive and dynamic domain structures is suggested to enhance the algorithm’s performance by refining and utilizing multiple swarm adaptive and fuzzy operators. The efficacy of the algorithm is verified through experiments with 13 popular TSP benchmark datasets, thereby demonstrating the complexity of the SFACO approach. Ultimately, the path planning problem of surface 3D laser scanning measurement is addressed by employing the proposed SFACO algorithm in conjunction with a nominal distance matrix.

## 1. Introduction

### 1.1. Background

With the rapid development of precision measurement technology in the manufacturing field, the traditional manual and CMM measurements for measuring intricate surfaces have been replaced by 3D laser scanning measurement. In addition, 3D laser scanning technology utilizes laser ranging, which involves scanning the object to be measured with line structured light and collecting point cloud data from the surface of the object to be measured [1]. After performing a series of mathematical calculations, a three-dimensional model of the object to be measured is created from point cloud data [2]. In the field of linear structured laser scanning technology, the calibration of a 3D laser scanning system, laser strip center extraction, laser scanning path planning, and point cloud data processing is essential for enhancing the 3D scanning process in terms of both quality and speed. The 3D scanning process necessitates laser scanning path planning and attitude confirmation, which involves processing the 3D point cloud of the object being tested to identify the sampling points. Moreover, based on the sampling points, the scanning range and scanning direction is important for constructing a laser scanning attitude model so as to ultimately generate a full scanning path [3]. In order to obtain a comprehensive 3D model and enhance the scanning accuracy and speed, it is imperative to plan various attitude states to form the best scanning position and angle. Furthermore, laser scanning sensors possess a high degree of precision and swiftness; however, there is also the potential for a collision between the sensor and the workpiece, as well as the need for multiple scans when measuring intricate surfaces (which can lead to an inefficient use of resources). Consequently, it is essential to devise suitable algorithms to map out the laser probe’s scanning path in order to achieve the most effective path plan, thereby enhancing the scanning efficiency and shortening the scanning time through successful obstacle avoidance. Recently, the exploration of 3D scanning path planning for intricate surfaces has been a major focus of research in the field of 3D scanning techniques. In 2023, Sedao et al. [4] obtained a 3D point cloud from the surface of the object to be measured, identified the points to be measured by a series of target points centered on a 3D mesh that was tailored to the curvature of the surface, and utilized surface discovery computation to determine the position of the laser probe in the area, which was performed so as to carry out the grid cell laser texturing step by step to acquire the ideal scanning path. Diao et al. [5] combined a point cloud slicing algorithm with a curve fitting algorithm to map out the machining path of intricate castings, as well as suggested a novel cross-source point cloud alignment technique, which drastically enhanced the uniformity of the surface profile and the efficiency of the machining paths produced by the method. In 2022, Zong et al. [6] suggested an automated path planning 3D scanning system based on the raster projection principle to create a 3D scanning sensor. Zong suggested an algorithm for path planning that is based on the concept of virtual 3D scanning, which uses the CAD model of the scanning object to determine the most efficient scanning path. Huang et al. [7] employed discrete and continuous optimization of scanning paths by solving the TSP problem to compute the topology maps in order to determine dynamic scanning targets and paths. Yan et al. [8] suggested a two-part scanning and detection planning approach to address the scan line distribution and detection path planning issue. To begin with, an adaptive sampling strategy (CLS) resembling a spiderweb is created to detect shape errors by arranging scan lines in a spiderweb shape pattern. Next, a detection path planner is employed to map out both local and global detection routes. Moreover, a novel ant colony optimization algorithm (IACO) has been suggested to create time-efficient routes for discrete scan lines, thereby enhancing the scanning probe’s inspection capacity on a CMM.

The process of path planning involves discovering the most efficient route that links the beginning and end points while adhering to certain restrictions. In the process of 3D laser scanning intricate surfaces, the laser probe traverses all the measured points in a specific sequence. Optimizing the sequence of points to be measured decreases the time it takes for the laser probe to move, as well as help it to avoid obstacles so as to prevent multiple measurements. The essence of the laser scanning path planning problem lies in the order in which the blade profile is scanned. Therefore, the laser probe path planning problem that follows intricate surface slicing can be conceptualized as a combinatorial optimization problem, namely the TSP problem, which is the shortest path planning problem and a traditional combinatorial optimization problem. The TSP is one of the most researched problems in optimization and is often regarded as a benchmark problem for several optimization methods [9]. It has been demonstrated that the TSP problem is NP-hard, thus making it impossible to find algorithms that are exact in polynomial time. The most common solutions to the TSP problem are heuristic algorithms, meta-heuristic algorithms, and linear programming. Meta-heuristic algorithms include genetic algorithms [10], ant colony algorithms [11], particle swarm optimization algorithms [12], cuckoo research [13], etc. There have been many studies and applications on the TSP problem in recent years. Zhou et al. [14] proposed a novel ACO approach and designed an innovative adaptive learning mechanism (ADACO). The ACO algorithm for TSP was modeled under the reinforcement learning framework, and the optimal policy was learned via a stochastic gradient descent approach. Subsequently, the ACO algorithm incorporates the adaptive gradient descent strategy under the name ADACO. Zhou’s proposed strategy can make use of the update history of each dimensional pheromone to achieve intelligent convergence. Reda et al. [15] used a newly proposed discrete variant of the cuckoo search algorithm to solve the OPR problem. Five modifications were made to the discrete cuckoo search algorithm before applying it to the traditional TSP problem. Zhang et al. [16] proposed a discrete cuckoo search algorithm based on random wandering and cluster analysis for the TSP. For the large-scale TSP problem, a k-means algorithm was used to divide the cities into k classes for optimization, and then a stochastic algorithm was used to combine them. A simple 2-opt operator was utilized as a local optimization operator to accelerate the convergence of the algorithm. The TSP problem has a wide range of applications, including robot path planning [15], cutting path optimization [16], and automatic scanning path planning for complex surfaces [8].

### 1.2. Ant Colony Optimization

Ant colony optimization (ACO) is a meta-heuristic algorithm designed and popularized by Dorigo; it allows ants to determine the direction of their journey by gauging the pheromone concentration. The greater the concentration of pheromones, the more likely they are to follow and crawl the path. Consequently, the most efficient route has the greatest pheromone concentration. The key to the ACO approach is the selection of nodes and the updating of pheromones. Due to its design principle, the ACO algorithm is highly regarded for its ability to solve a variety of combinatorial optimization problems. Pal et al. [17] suggested an ant colony optimization algorithm that incorporates a fuzzy logic-based contingency ranking index for the transmission and contact lines of a multi-region grid; furthermore, an enhanced ant colony algorithm was employed to reduce transmission active and reactive power losses during contingencies, thus augmenting power flow through other transmission lines. A two-tier multi-objective path planning model was proposed by Sui et al. [18] with the objective of minimizing path length and hazard distance. The algorithm utilizes an ant colony optimization algorithm to create a series of tasks for the upper model in the outer layer and then creates a two-layer hybrid algorithm. Chuang et al. [19] suggested a real-time two-stage ant colony (RTACO) algorithm to reduce the strain on the task offloading algorithm and create a dependable, high-performance edge computing system. Rivera et al. [20] suggested HyperACO, a hyper-heuristic algorithm that seeks to identify the optimal combination of multiple interval ranking models that are embedded in MOEA to address a multi-objective optimization issue. HyperACO not only has the ability to choose the most suitable model, but also to combine existing models to accurately address a particular multi-objective optimization problem. The field of intelligent algorithm research has been abuzz with discussions on how to enhance the ACO approach to address its weaknesses. Das et al. [21] suggested a quantum-inspired ant colony optimization (Qi-ACO) algorithm, which is based on quantum-inspired ant colony optimization algorithms in order to address a sustainable four-dimensional travel quotient problem (4DTSP). Quantum-inspired techniques enable rapid computation and the defuzzification of type-2 fuzzy variables through the critical value (CV) method, which is essential for the initialization and updating of pheromones in Qi-ACO. Liu et al. [22] suggested a novel version of the ACO algorithm with adjustable pheromone concentration settings, a heuristic mechanism with directional discernment, an enhanced pseudo-random transfer strategy, and a pheromone. Furthermore, an upgraded ACO approach (IHMACO) was created with improved tactics such as dynamic alteration of the evaporation rate. Ren et al. [23] designed a new improved ACO optimization algorithm to solve the model, using pheromone and heuristic algorithms to generate an initial solution to ensure the quality of the initial population. A forbidden search operator containing five neighborhood operators was constructed to improve the local search capability of the algorithm, and a simulated annealing mechanism was introduced to update the global pheromone to increase the diversity of the population. Pu et al. [24] suggested a novel fractional order ACO (FACA) algorithm, which is based on the cooperative learning approach of fractional long-term memory. The integer order ACO algorithm’s transformation behavior is altered by utilizing the inherent power of fractional algorithms, which is achieved by substituting straightforward one-step probabilities with more intricate fractional derivatives that include some forward-looking information. For the study of complex surface three-dimensional laser scanning path planning problems, the laser scanning sensor measurement should prioritize accuracy and speed, yet it is accompanied by exorbitant scanning costs and the wasteful utilization of resources, as well as other phenomena in complex surface measurements. Consequently, it is essential to devise suitable algorithms to map out the laser probe’s scanning path in order to obtain the most efficient path plan, thereby enhancing the scanning efficiency and shortening the scanning time through successful obstacle avoidance. Consequently, this research will utilize the enhanced ant colony algorithm to devise a plan for measuring 3D surfaces, thus allowing the line laser to measure along the most efficient route and acquire the most precise measurement data of 3D surfaces with greater efficiency.

### 1.3. Contributions of This Manuscript

In this manuscript, three coordinate systems are established based on the motion model of laser scanning on a four-coordinate laser scanning measuring machine to tackle the issue of 3D laser scanning path planning for complex surfaces. The number of readings of the axes of the measuring machine located at the point to be measured under the optimal measuring attitude was reversed and solved from the transfer matrix of the transformation relationship between the coordinate systems. Afterward, the nominal distance matrix between the points to be measured was established based on the readings of each axis of the measuring machine of the points to be measured in the optimal measuring attitude. Subsequently, a strategy involving sub-populations and adaptive heuristic factors was devised to enhance the ACO approach, while a dynamic domain structure was employed to augment the diversity of the ACO population. An adaptive ant colony algorithm with sub-population and fuzzy logic (SFACO) was created. Ultimately, the SFACO approach was utilized to address the intricate surface 3D laser scanning path planning issue by replacing the Euclidean distance with the nominal distance at the optimal measurement attitude for calculating the fitness value. The TSP benchmark dataset was used to assess the accuracy of the SFACO approach in the experiments, and the SFACO algorithm was then employed to simulate 3D laser scanning path planning for intricate surfaces in order to determine the most efficient scanning path.

The contributions of this manuscript are as follows:

- This manuscript establishes three coordinate systems based on a laser scanning four-coordinate measuring machine. Moreover, it inversely solves the machine readings of each axis of the object to be measured in normal measuring attitude according to the conversion between coordinate systems. Furthermore, we constructed the nominal distance matrix by utilizing the machine readings of each axis of all the points to be measured on the object to be measured in the optimal measuring attitude.
- An adaptive ant colony with sub-population algorithm was designed. Ant colonies can simulate human social learning through sub-population and adaptive parameter strategies, as well as improve the convergence performance of ant populations by the guidance of transcendental knowledge.
- A 3-opt neighborhood structure was implemented to alter the course of the ant colony in order to enhance the variety of the population. The algorithm’s guidance for optimizing the next-generation population was enhanced by applying a fuzzy logic strategy to dynamically adjust pheromone volatilization parameters. The TSP benchmark test confirmed the effectiveness of the proposed SFACO algorithm.
- The proposed SFACO algorithm is utilized to identify the most efficient planning path for the intricate 3D laser scanning path planning problem.

The rest of the manuscript is structured as follows: Section 2 describes the design of the laser measurement pose, whereby the specific process of constructing the nominal distance matrix through the establishment, the transformation of the coordinate system, and the establishment of the normal vector measurement pose are focusing upon. Section 3 focuses on the improvement and innovation strategies of the various parts of the algorithm of the adaptive ACO approach with sub-population and fuzzy logic, as well as on the overall framework of the SFACO algorithm. Section 4 is devoted to experiments and analysis, including TSP benchmarking and surface 3D laser scanning path simulation experiments. Finally, Section 5 summarizes the work that was conducted.

## 2. The Design of the Laser Measurement Attitude

### 2.1. Establishing and Converting the Coordinate System

**Figure 1:**
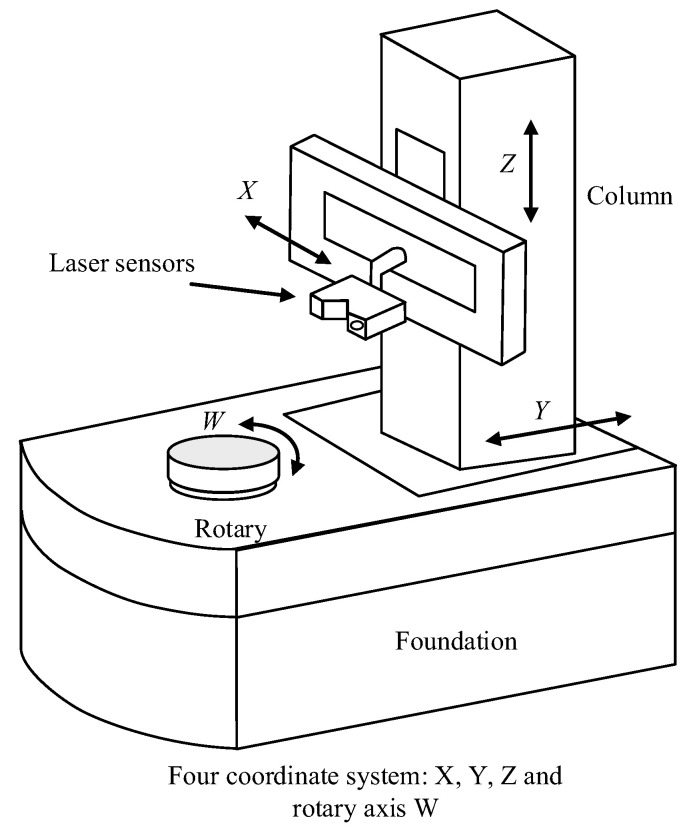
Schematic diagram of the four-coordinate laser scanning measurement.

According to the measurement requirements of complex curved parts, the laser scanning four-coordinate measuring system, as shown in Figure 1, was the four-coordinate laser scanning measuring machine model used in this paper. The main body of the measuring machine was made up of three vertical axes, X−Y−Z, and a rotary table *W*. Moreover, the laser sensor was fixed in the *Z* axis column and the part to be measured was fixed on the rotary table *W*. Three coordinate systems were established based on the configuration of the measuring machine system: the measuring machine motion coordinate system $O_{M}$, the laser measurement coordinate system $O_{L}$, and the workpiece coordinate system $O_{P}$.

The measuring machine coordinate system $O_{M}$ was fixed to the rotary table center as the origin *O*, and the coordinate system axis xyz and measuring machine movement axis XYZ were parallel. The workpiece coordinate system $O_{P}$ was fixed on the rotary table to the rotary table center as the origin *O*, and this was set when the measuring machine *W*-axis readings were at time 0; in addition, the workpiece coordinate system and the measuring machine coordinate axis XYZ direction were consistent. The laser measurement coordinate system $O_{L}$ was established on the *Z*-axis column of the measuring machine, and the three coordinate axes of the coordinate system were, respectively, parallel to the XYZ movement axis of the measuring machine, with the intersection of the mounting position of the laser sensor and the *Z*-axis column as the origin *O*.

Let there be a point *Q* in the measurement space, whose coordinates on the coordinate systems $O_{M}$, $O_{L}$, and $O_{P}$ are denoted as $q_{M}$, $q_{L}$, and $q_{P}$, respectively. In addition, let the transformation matrix between $O_{M}$ and $O_{L}$ be $G_{ML}$, between $O_{M}$ and $O_{P}$ be $G_{MP}$, and between $O_{P}$ and $O_{L}$ be $G_{PL}$. Then, the transformation relationship between the coordinate systems can be expressed as Equation (1):

(1) $$\left\{\begin{array}{c} q_{M}=G_{ML}\cdot q_{L}\\ q_{M}=G_{MP}\cdot q_{P}\\ q_{P}=G_{PL}\cdot q_{L} \end{array}\right..$$

While the measuring machine is functioning, let a point on the workpiece be measured such that it has each motion parameter $\left(\theta_{x},\theta_{y},\theta_{z},\theta_{w}\right)$ in the measuring machine system. Furthermore, let this be such that each transformation matrix can be, respectively, expressed as Equations (2) and (3).

(2) $$G_{ML}=\left[\begin{array}{cccc} 1 & 0 & 0 & \theta_{x}\\ 0 & 1 & 0 & \theta_{y}\\ 0 & 0 & 1 & \theta_{z}\\ 0 & 0 & 0 & 1 \end{array}\right],$$

(3) $$G_{MP}=\left[\begin{array}{cccc} \cos\theta_{w} & -\sin\theta_{w} & 0 & 0\\ \sin\theta_{w} & \cos\theta_{w} & 0 & 0\\ 0 & 0 & 1 & 0\\ 0 & 0 & 0 & 1 \end{array}\right].$$

Therefore, $G_{PL}$ can be obtained from Equations (1)–(3), as in Equation (4), as follows:

(4) $$G_{PL}=G_{MP}^{-1}\cdot G_{ML}=\left[\begin{array}{cccc} \cos\theta_{w} & \sin\theta_{w} & 0 & \theta_{x}\cdot \cos\theta_{w}+\theta_{y}\cdot \sin\theta_{w}\\ -\sin\theta_{w} & \cos\theta_{w} & 0 & -\theta_{x}\cdot \sin\theta_{w}+\theta_{y}\cdot \cos\theta_{w}\\ 0 & 0 & 1 & \theta_{z}\\ 0 & 0 & 0 & 1 \end{array}\right].$$

### 2.2. Normal Measurement Attitude Establishment

**Figure 2:**
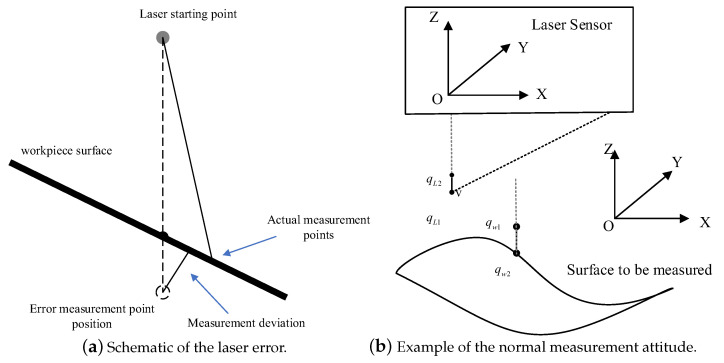
Attitude measurement in the normal direction.

Figure 2a is a schematic diagram of the laser error. The laser error diagram reveals that the laser emission point is not directly beneath the actual measurement point during laser scanning. As the angle of inclination between the workpiece and the laser incident point increases, the measurement deviation, which is the distance between the error measurement point and the surface of the workpiece, also rises. Consequently, the measurement accuracy is highest when the incident laser is perpendicular to the workpiece surface. The normal measurement attitude schematic is shown in Figure 2b. The optimal location for measuring the laser sensor is the midpoint of the sensor range, and the midpoint of the range from the *Z*-axis column distance is set at *m*. Then, the ideal position of the point to be measured in the laser measurement coordinate system is $q_{L1}=\left(0,-m,0\right)$, and the laser ray on the distance from the point of unit length of $q_{L1}$ is $q_{L2}=\left(0,-m+1,0\right)$. Set the part to be measured on a point that is to be measured $q_{P1}$, which is located in the workpiece coordinate system. Let $q_{P1}=\left(x,y,z\right)$, $\left(n_{x},n_{y},n_{z}\right)$ be the point to be measured $q_{P1}$, and let it be normal to the direction of the unit vector. Therefore, the point $q_{P2}=\left(x+n_{x},y+n_{y},z+n_{z}\right)$ is in the direction that is normal to the point to be measured at a distance of the unit length from the point that can be obtained.

Since the highest measurement accuracy is achieved when the incident laser is perpendicular to the surface of the object to be measured, as well as when the laser sensor measuring point vector $\vec{q_{L1}q_{L2}}$ overlaps with the laser sensor measure point normal vector $\vec{q_{P1}q_{P2}}$ of the object to be measured, then the measuring instrument is in the optimal measuring position. According to Equation (1), it is possible to relate $q_{P}$ with $q_{L}$ through the transformation matrix as in Equations (5) and (6):

(5) $$\left\{\begin{array}{c} q_{P1}=G_{PL}\cdot q_{L1}\\ q_{P2}=G_{PL}\cdot q_{L2} \end{array}\right.,$$

(6) $$q_{P2}-q_{P1}=G_{PL}\cdot \left(q_{L2}-q_{L1}\right).$$

Substituting Equation (4) into Equations (5) and (6) yields Equations (7) and (8) as follows:

(7) $$\left\{\begin{array}{c} n_{x}=\sin\theta_{w}\\ n_{y}=\cos\theta_{w} \end{array}\right.,$$

(8) $$\left\{\begin{array}{c} x=\theta_{x}\cdot \cos\theta_{w}+\left(\theta_{y}-m\right)\cdot \sin\theta_{w}\\ y=-\theta_{x}\cdot \sin\theta_{w}+\left(\theta_{y}-m\right)\cdot \cos\theta_{w}\\ z=\theta_{z} \end{array}\right..$$

When setting $\theta_{w}\in \left[0^{\circ },360^{\circ }\right]$, based on Equations (7) and (8), as well as the point to be measured $\left(x,y,z\right)$ and the normal vector unit vector $\left(n_{x},n_{y},n_{z}\right)$, then the parameter $\left(\theta_{x},\theta_{y},\theta_{z},\theta_{w}\right)$ of the measuring machine system can be obtained under the optimal measuring attitude.

### 2.3. Nominal Distance Matrix Construction

The laser sensor will traverse all the points to be measured from the normal direction in a certain order so as to expedite the measurement process and reduce the amount of resources wasted through repetitive scanning. An enhanced ant colony algorithm was employed for the blade profile measurement path planning, which was performed to refine the order of the points to be measured and to ensure the line laser measures along the most efficient path, thus obtaining the precise measurement data of the complex surface with greater efficiency. In this paper, the normal measurement attitude was used to map the points to the independent motion axes of the measuring machine under the optimal attitude. The nominal distance matrix between the points to be measured was then constructed with practical measurement significance, and the nominal distance matrix was then substituted into the improved ant colony algorithm to optimize the measurement order of the laser sensor among the points to be measured.

According to Equations (7) and (8), the measurement machine’s motion distance between the two points to be measured was converted into the individual motion distance of each axis, and the measurement attitude of the two to-be-measured points was, respectively, set as $\left(\theta_{x1},\theta_{y1},\theta_{z1},\theta_{w1}\right)$ and $\left(\theta_{x2},\theta_{y2},\theta_{z2},\theta_{w2}\right)$ while determining the vector of the motion distance of the measuring machine between the two points, as in Equation (9):

(9) $$\vec{d}=\left(\left|\theta_{x1}-\theta_{x2}\right|,\left|\theta_{y1}-\theta_{y2}\right|,\left|\theta_{z1}-\theta_{z2}\right|,\left|\theta_{w1}-\theta_{w2}\right|\right).$$

In Equation (9), xyz is the moving axis and *w* is the rotary axis. Due to the disparity in units between the two axes, the mere distance of movement did not accurately depict the temporal disparity between the two points. Through the standardization of motion gap characteristics, the distance of this switching motion was determined by selecting the maximum distance motion axis, thereby enabling the definition of the nominal distance between the two points to be measured as Equation (10):

(10) $$d_{1,2}=\max\left(Norm\left(\left|\theta_{x1}-\theta_{x2}\right|,\left|\theta_{y1}-\theta_{y2}\right|,\left|\theta_{z1}-\theta_{z2}\right|,\left|\theta_{w1}-\theta_{w2}\right|\right)\right),$$

where Norm is denoted as *z*-score normalized and calculated as $X=\frac{X_{i}-\mu }{\sigma }$, and μ and σ are the mean and variance of the distance difference of all axes, respectively. Consequently, based on the nominal distance between the two points to be measured in Equation (10), the nominal distance matrix between each neighboring point to be measured can be constructed as Equation (11):

(11) $$D=\left[\begin{array}{cccc} d_{1,1} & d_{1,2} & \cdots & d_{1,n}\\ & d_{2,2} & \cdots & d_{2,n}\\ & & \ddots & \vdots \\ & & & d_{n,n} \end{array}\right].$$

Let the traversal order between *n* to-be-measured points be $\left\{r_{1},r_{2},\cdots ,r_{n}\right\}$; thus, the total nominal distance after traversing all to-be-measured points is obtained according to Equation (11), as in Equation (12), as follows:

(12) $$f=\sum_{i=1}^{n-1}d_{r_{i},r_{i+1}}.$$

This paper utilizes the SFACO algorithm for path planning, with the nominal distance serving as the optimization objective. Consequently, the fitness function is formulated as per Equation (12) in this particular problem.

## 3. Adaptive Ant Colony with Sub-Population and Fuzzy Logic (SFACO) Algorithm

This manuscript proposes an ant colony algorithm based on adaptive learning strategies and fuzzy logic, and it combines dynamic fields with fuzzy logic control to imitate human social learning. By utilizing population diversity calculations and transcendental knowledge, the algorithm partitions population strategies, incorporates adaptive heuristic factors according to population diversity, and enhances the convergence performance of ant populations. These steps were performed to prevent a decline in the population diversity during the later stages of the algorithm, where the ant colony path undergoes reprogramming with a dynamic neighborhood structure. Fuzzy logic control was employed to modify the algorithm parameters and enhance the algorithm’s performance, thereby optimizing the guidance of the previous generation population search results for the next generation population search process.

### 3.1. Population Diversity

Currently, there is a great deal of research into how population diversity is represented in population intelligence algorithms, with many techniques utilizing Euclidean distance representation or algorithm fitness value to illustrate population diversity [25]. The fitness of the algorithm in the ant colony algorithm is essentially determined by the distance covered by each ant to complete all the points; thus, this paper was also formulated according to the algorithm’s fitness value in the representation of population diversity, as shown in Equation (13):

(13) $$\left\{\begin{array}{c} \bar{f}=\frac{1}{N}\sum_{i=1}^{N}f_{i}\\ pD_{i}=Nor\left(\frac{1}{N}\sum_{i=1}^{N}\left|f_{i}-\bar{f}\right|\right)\\ pD=\sum_{i=1}^{N}\varpi_{i}pD_{i}, \end{array}\right.$$

where *N* is the number of ants in the colony, $f_{i}$ is the fitness value of the *i*-th ant, $pD_{i}$ is the difference between the fitness value of each ant and the average fitness value, Nor denotes the normalization calculation, and pD denotes the overall diversity of the population. Moreover, $\varpi_{i}$ is usually taken as $\frac{1}{N}$.

### 3.2. Sub-Population Strategies

The utilization of sub-population strategies is commonplace in intelligent algorithms. In studies based on sub-population strategies, the division of populations and the size of each sub-population are essential for preserving the variety of the individuals in the algorithm. Nevertheless, the majority of current sub-population strategies for sub-population division rely on determining the quantity of individuals within the sub-population and dividing them based on individual spacing rather than adapting to the dynamic fluctuations of all individuals in the population. Consequently, to enhance the flexibility of the sub-population strategies for the ACO algorithm, this section utilizes population division according to the fitness value to adjust the population division in real time through the fluctuating size of the fitness value, and the division is structured as shown in Equation (14) [26]:

(14) $$\begin{array}{c} f_{1}=pD_{\min}\\ f_{2}=pD_{\min}+\frac{1}{3}\left(pD_{\max}-pD_{\min}\right)\\ f_{3}=pD_{\min}+\frac{2}{3}\left(pD_{\max}-pD_{\min}\right)\\ f_{4}=pD_{\max}\\ \Omega_{j}^{t}=\left\{\left.X_{i}^{t}\right|f_{j}\leq pD\left(X_{i}^{t}\right)<f_{j+1},i=1,2,\cdots N,\right\}j=1,2,3, \end{array}$$

where $pD_{\min}$ and $pD_{\min}$ denote the minimum and maximum values of population diversity, respectively; $\left\{f_{j},j=1,2,3,4\right\}$ denotes the boundary of the sub-population; $X_{i}^{t}$ denotes the path length of the *i*-th ant in the *t*-th iteration; and $\Omega_{j}^{t}$ denotes the *j*-th sub-population in the *t*-th iteration. Three sub-populations of a balanced size were obtained by dividing the population into three equal parts.

The evolution of the ACO algorithm involves two crucial stages: probabilistic selection and pheromone updating. The probabilistic selection formula involves the consideration of the path length and pheromone concentration. In this section of adaptive ant colony optimization with sub-population and fuzzy logic, the initial knowledge is computed utilizing the probabilistic selection formula shared by ACO algorithms, thereby ensuring that the node with the greatest likelihood is chosen in every path node selection. The probability selection formula was used to determine the predetermined route of all the ants. After the a priori path scheme was calculated, the diversity of the population was calculated according to the diversity shown in Equation (14). The population was then divided according to Equation (14) to obtain three sub-populations of a balanced size.

### 3.3. Adaptive Heuristic Factor

The ant colony algorithm’s success in adjusting the search direction hinges on its ability to calculate the transfer probability. The pheromone concentration and the value of the heuristic function between two nodes are crucial factors in the calculation of transfer probability. The transfer probability is significantly influenced by both the pheromone heuristic factor α and the expectation heuristic factor β when determining pheromone concentration and heuristic function. The pheromone heuristic factor α indicates the significance of the amount of data the ants accumulate while moving in order to direct the search of their colonies. If α is too high, the ants will likely opt for the paths they have previously traversed, thus diminishing the randomness of the search; conversely, if its value is too low, the search will be too early to reach the local optimum. The heuristic function factor β indicates the significance of the heuristic information in directing the ant colony search process, while its magnitude signifies the potency of the a priori and deterministic factors in the ant colony search process. When it is too large, even though the rate of convergence will be hastened, it is easy to become trapped in the local optimum; when it is too small, it is easy to be caught up in the random search and unable to find the best solution. Consequently, this section suggests a transfer probability updating rule that takes into account sub-population adaptive heuristic factors in order to strike a balance between exploration and exploitation capabilities, and this is achieved by replicating human social learning capabilities.

To replicate human social learning intelligence, the ant population was divided into three distinct sub-populations through a sub-population strategy based on the evolutionary level of ants in each iteration, with pD_G representing the global ideal ant diversity among all the populations and pD_S representing the optimal ant diversity among the sub-populations. Furthermore, the common ants that remained to reach the average diversity of the populations were calculated, and the heuristic factors for all the individuals mentioned above were formulated in the following Equations (15) and (16):

(15) $$\varpi_{i}^{t}=\left\{\begin{array}{cc} \frac{\frac{\sum_{j=1}^{N\left(t\right)-1}pD\_S_{j}^{t}}{N\left(t\right)-1}}{pD\left(X_{i}^{t}\right)} & ifX_{i}^{t}=pD\_G^{t}\;\\ \frac{\frac{pD\_S_{j+1}^{t}}{N\left(t\right)-1}}{pD\left(X_{i}^{t}\right)} & ifX_{i}^{t}=pD\_S^{t}\;\\ \frac{pD\_S_{S_{\left(i\right)}^{t}}^{t}}{pD\left(X_{i}^{t}\right)} & else, \end{array}\right.$$

(16) $$\left\{\begin{array}{c} \alpha =1+3*F\left(\varpi_{i}^{t}\right)\\ \beta =3+2*F\left(\varpi_{i}^{t}\right) \end{array}\right.,$$

where $N\left(t\right)$ denotes the number of sub-populations, $pD\_S_{j}^{t}$ denotes the diversity value of the best ant of the first *j* sub-population at the *t*-th iteration, and $pD\_G^{t}$ denotes the diversity value of the globally optimal ant. Moreover, $S_{\left(i\right)}^{t}$ denotes the sub-population where the *i*-th ant is located at the *t*-th iteration, and $F\left(\varpi_{i}^{t}\right)$ is the cumulative distribution function of a positive tautological distribution, where ants with higher diversity values tend to be weighted more heavily and are guaranteed to be $\alpha \in \left[1,4\right]$, $\beta \in \left[3,5\right]$ under this strategy.

### 3.4. Dynamic Neighborhood Structures

The classical ACO algorithm relies on the concentration of pheromones and the distance between nodes to find the most suitable paths, which often results in the ant colony paths converging in the later stages of the algorithm, thus reducing the diversity of the population and making it more likely for it to become stuck at the local extremes. Consequently, the SFACO algorithm utilizes a dynamic 3-opt neighborhood structure to modify the ant colony paths in order to enhance population diversity [27].

**Figure 3:**
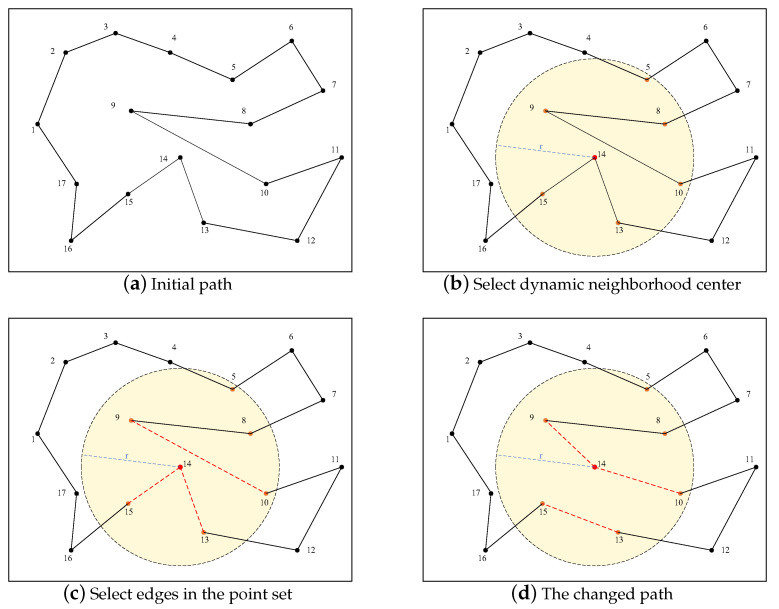
The domain selection was exemplified by 3-opt exchanges. (The example graph selects point 14 (red point) as the center point of the dynamic neighborhood, constructs the set of cities (all orange points) based on the radius r, and randomly selects the edges (red dashed lines) for the transformation, and the graph is d after the transformation).

The 3-opt algorithm is a neighborhood structure with multiple variations, and it is a local search method often applied to TSP problems. The 3-opt algorithm performs a local search to generate a new feasible solution in the neighborhood, which is also where the ACO algorithm provides a feasible solution. The principle of the 3-opt algorithm is to select three sub-paths in the paths, remove them from the paths of the ACO algorithm, and then try to reconnect them to different paths in a different way. By establishing connections, this procedure creates fresh and diverse routes, as well as acquires multiple potential routes. As the algorithm progresses to the later stage, the 3-opt randomized path selection will lead to an inadequate and subpar set of potential candidates. The paths that are randomly shifted are longer than the original paths; as such, the probability that they will be selected as the current optimal paths is extremely low. This inferior set will result in a lot of unnecessary computations at the later stage of the iteration, which will increase the algorithm’s complexity. Hence, the SFACO algorithm utilizes a dynamic 3-opt neighborhood, as illustrated in Figure 3. Moreover, it exemplifies a straightforward instance of neighborhood selection through a 3-opt exchange, and Figure 3a represents the initial path. The center of the circle shown in Figure 3b was chosen at random from point 14, with *r* being triple the average path. Assume that the set of cities selected in this way consists of {5,8,9,10,13,14,15}. The edges $\left(9,10\right)$, $\left(13,14\right)$, and $\left(14,15\right)$ within the set of randomly selected points are shown in Figure 3c, which are the cut off and exchanged for paths. The result can be obtained as shown in Figure 3d. The connection with the shortest path length was selected as the new connection by calculating the total path length of the different connections. The 3-opt algorithm candidate set was created by randomly picking a node from the set of all nodes, whereby the points in its vicinity were calculated based on the radius. Then, the edges formed by certain points in the set of points were randomly picked to carry out the 3-opt algorithm candidate set establishment once the neighborhood point set was acquired. By computing the dynamic 3-opt neighborhood structure, the algorithm was able to limit the range of potential candidates in the later stages, thus decreasing the computational load of the algorithm.

### 3.5. Fuzzy Logic Control

**Figure 4:**
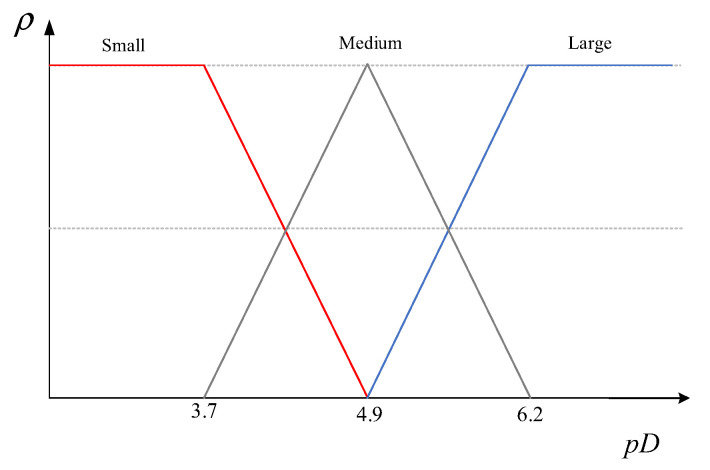
Fuzzy logic graphical representation of the pheromone volatilization factors.

The classical ACO algorithm takes into account the strength of the pheromone when selecting a path, while the pheromone updating strategy involves combining the pheromone-volatilized residuals from the previous generation with the pheromone concentration of the current newly generated pheromone, which is determined by the length of the path. Consequently, the volatilization intensity of the pheromone from the preceding generation significantly impacts the concentration of the pheromones in the ant colony, thereby potentially serving as a focal point for the algorithm’s convergence. Consequently, it is of utmost importance to determine the appropriate pheromone volatilization strength factor. In the classical ACO algorithm, the pheromone volatilization factor ρ is a constant value, which makes the pheromone volatilization intensity of the algorithm in each iteration consistent to the point where there is not enough guidance for the optimization of the next generation of populations; as such, it is essential to adjust the pheromone volatilization intensity judiciously during the execution of the algorithm. As previously stated, the algorithm utilizes the population fitness value to mirror the diversity of the population and illustrate the disparities among the individuals within the population. As illustrated in Figure 4 [28], population diversity was classified as “good”, “medium”, and “poor” during the course of population evolution. The pheromone volatilization parameter ρ was adjusted dynamically using fuzzy logic control during the evolutionary process to achieve a better utilization of the algorithm for the previous generation’s experience, and this was performed according to the performance of the population diversity. Consequently, this section puts forward a fuzzy logic rooted in population diversity for the purpose of updating the pheromone volatilization factor ρ.

Equation (13) represents the expression of population diversity, and the defuzzification method used in this section was based on population diversity. As such, the output of the fuzzy logic system can be defined as Equation (17):

(17) $$\left\{\begin{array}{cc} \rho_{i,j}^{t}=\frac{1}{T_{i,j}^{t}}\left[\rho_{i,j}^{t}+a+\frac{sin\left(pD_{k}\right)}{2\pi }\right] & ,T_{I,J}^{t}\neq 0\;\\ \rho_{i,j}^{t}=0.2 & ,others \end{array}\right.,$$

where $\rho_{i,j}^{t}$ denotes the pheromone volatilization intensity factor between the *t*-th generation of points $\left(i,j\right)$ to be measured, $pD_{k}$ denotes the diversity value of the *k*-th ant, and $T_{i,j}^{t}$ denotes the number of times the pheromone volatilization intensity is updated between the *t*-th generation of points $\left(i,j\right)$ to be measured. When $pD_{k}$ is evaluated as “poor”, it is a=0.1; when it is evaluated as “medium”, then it is a=0.2; and when it is evaluated as “good”, then it is a=0.3.

### 3.6. SFACO Algorithm Steps

**Figure 5:**
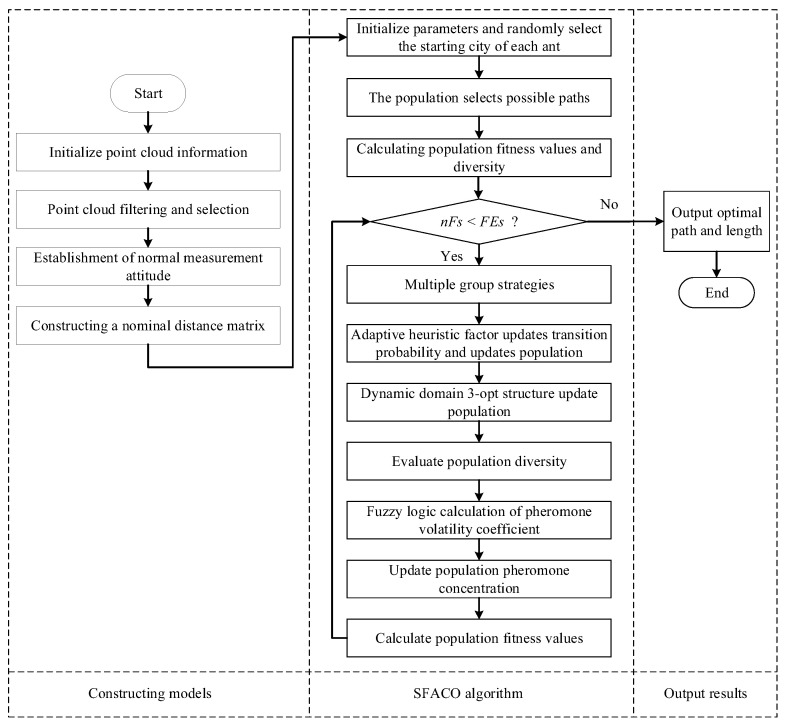
SFACO algorithm flowchart.

The SFACO algorithm proposed in this manuscript, as illustrated in Figure 5, was based on the above algorithmic improvements. This was shown, along with its application flowchart, in the laser scanning path planning.

## 4. Simulation Experiment Verification

In order to verify the performance of the proposed algorithm, the algorithm was validated from two parts, namely the TSP simulation experiment based on the SFACO algorithm and the laser scanning surface path planning simulation experiment that was also based on the SFACO approach.

### 4.1. TSP Simulation Experiment Based on the SFACO Approach

In this section, the test results of the SFACO approach on the benchmark dataset of TSP are mainly discussed. TSP is a typical discrete combinatorial optimization problem, and it is often used for evaluating the search optimization ability of optimization algorithms. In this part of the experiment, the algorithm was mainly tested using the commonly used datasets obtained from TSPLIB [29]; the standard test set for TSP problems at the University of Heidelberg; and the datasets att48, berlin52, ch150, eil01, kroC100, lin105, pr76, pr144, rat99, rd100, st70, u159 and tsp225 were mainly selected for experiments. The number of nodes in each dataset was 48, 52, 150, 101, 100, 105, 76, 144, 99, 100, 70, 159, and 225, and their theoretical optimal values were 33,522, 7542, 6528, 629, 20,749, 14,379, 108,159, 58,537, 1211, 7910, 675, 42,080, and 3916, respectively.

In this part of the experiments, comparative experiments were conducted by using several state-of-the-art optimization algorithms in order to analyze the performance of the SFACO algorithm. The genetic algorithm (GA) [30], evolutionary simulated annealing algorithm (ESA) [31], fractional order particle swarm optimization (FPSO) [32], improved bat algorithm (IBA) [33], hybrid symbiotic organisms search (SOS), ACO algorithm (SOS–ACO) [34], and maximum and minimum ant colony algorithm (MMAS) [35] approaches were mainly used in the experimental environment of 16 GB RAM, 12th Gen Intel(R) Core(TM) (ASUSTeK COMPUTER INC., China) i5-12500H 2.50 GHz, and MATLAB R2020a. Under these conditions, 10 experimental comparisons were randomly executed with the number of iterations set to 200. The parameter settings of the ACO and SFACO approaches are shown in Table 1.

Table 2 shows the performance evaluation of several algorithms for common TSP problems. Table 2 mainly shows the performance of the 12 datasets (att48, berlin52, ch150, eil01, kroC100, lin105, pr76, pr144, rat99, st70, u159, and tsp225) when the GA, ESA, FPSO, IBA, MMAS, ACO, and the proposed SFACO approaches were used on them in this paper. The table, respectively, shows the mean, optimum, and standard deviation of the results obtained from each algorithm for ten experimental computations of the datasets. From the data recorded in Table 2, the optimal search ability of the basic ACO approach was relatively poor in all the datasets listed in the table, and the average path and shortest path values calculated by the ACO approach were significantly higher than the other optimization methods. In addition, the results of GA were significantly worse than the other algorithms, including ACO. Secondly, the MMAS and FPSO algorithms had relatively good optimal search ability and could obtain theoretical optimal results in the att48, berlin52, ch150, kroC100, lin105, pr76, and pr144 optimal searches. For the eil101 dataset, the MMAS and SFACO approaches were able to obtain the theoretical optimum, but several other algorithms fell short of the computational results of these two methods. The SFACO approach proposed in this paper was able to achieve the theoretical optimum in ten of the TSP datasets (att48, berlin52, eil101, kroC100, lin105, pr76, pr144, rat99, st70, and u159), and the FPSO calculation was found in the ch150 and tsp225 datasets. The effect was better, but most of the standard deviations of the SFACO calculation results were smaller than those of several other algorithms, which showed the better algorithmic stability of the SFACO approach. From the calculation results of the mean value, the mean value of the test results of the SFACO algorithm proposed in this paper on the berlin52 and st70 datasets were able to reach the theoretical optimum. In addition, the SFACO approach was tested on a total of 13 datasets, and the mean value of the test results of 6 of the datasets was smaller than that of the other algorithms, which also proves that the mean value of the test results of proposed SFACO approach is better than that of the other algorithms. Moreover, this also proved the stability of the algorithm and SFACO’s optimal search capability.

**Figure 6:**
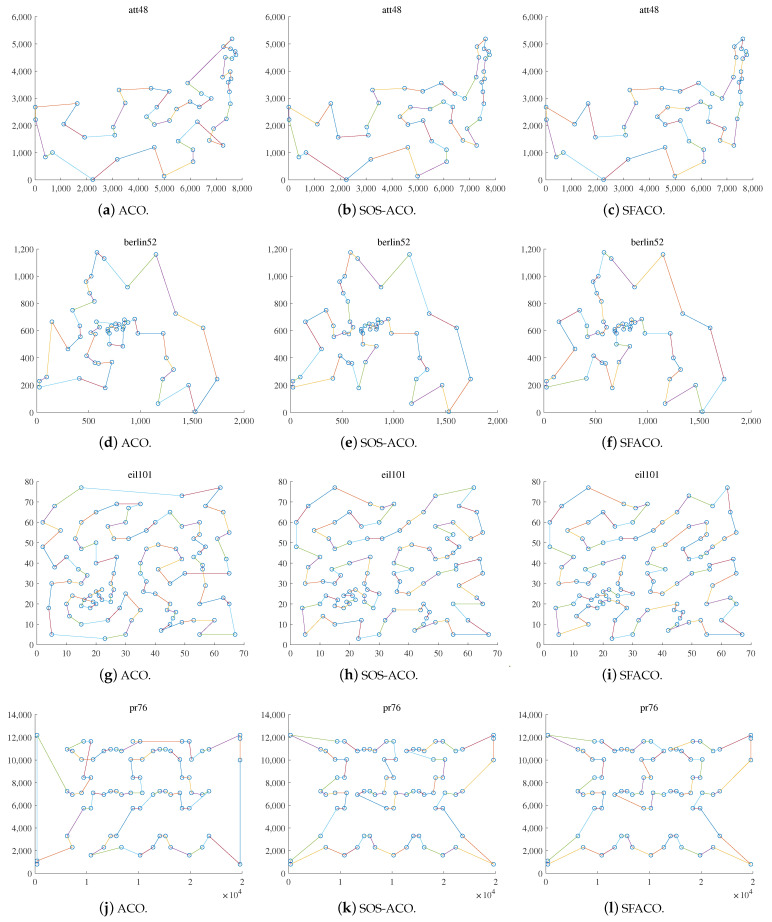
Comparisons of the ACO, SOS-ACO, and SFACO results in the TSP problem.

**Figure 7:**
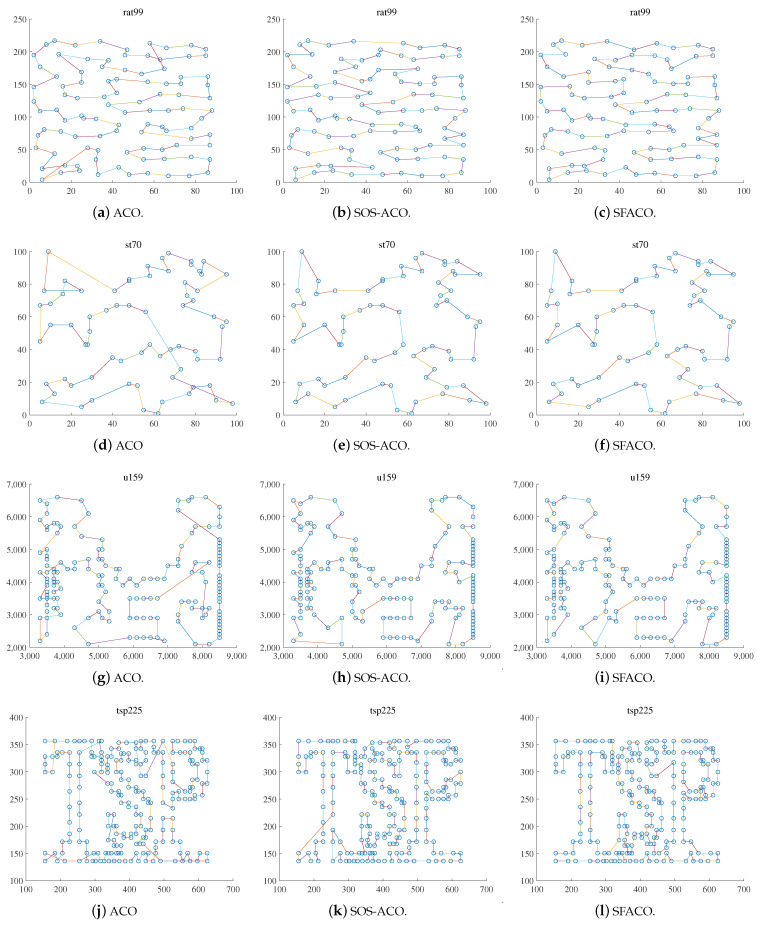
Comparisons of the ACO, SOS-ACO, and SFACO results in the TSP problem.

Further, in order to express the algorithm search capability more clearly, some of the test data in the TSPLIB dataset were selected to show and compare the results from the att48, berlin52, eil101, pr76, rat99, st70, u159, and tsp225 datasets, which were implemented as shown in Figure 6 and Figure 7.

Figure 6 and Figure 7a,d,g,j show the optimal path result plots of the att48, berlin52, eil101, pr76, rat99, st70, u159, and tsp225 datasets when using the basic ACO algorithm, respectively. Figure 6 and Figure 7b,e,h,k are the optimal path result plots when using SOS-ACO algorithm. Figure 6 and Figure 7c,f,i,l are the optimal path result plots when using the SFACO algorithm. The results of the basic ACO algorithm to compute att48, berlin52, st70, pr76, eil101, u159, and tsp225 were 34,845.63596, 7933.379828, 727.319193508222, 121,710.560462624, 692.073972654823, 45,793.5195407362, 4246.602961659, respectively. Meanwhile, the results for the datasets that were computed using the SFACO algorithm were 33,522, 7542, 675, 108,159, 635, 42,080, and 3985. In the att48, berlin52, st70, and u159 datasets, the solutions computed by the SFACO approach were found to be the same as their theoretical optimal solutions, while the absolute errors between the SFACO approach when based on the eil101 and tsp225 datasets, as well as their corresponding theoretical global optimums, were 6 and 69, respectively. Furthermore, it can be observed from Figure 6 and Figure 7 that, for the att48 and berlin52 test datasets, the nodes were more uniformly geometrically distributed within their neighborhoods. As in Figure 6a–c, the distribution density in the upper right corner of the graph for the att48 dataset was larger than that of the nodes in the lower left corner. The distribution density of nodes in the center region of the image of the berlin52 dataset, as shown in Figure 6d–f, was larger than that of nodes in the surrounding region, and the relative distances between any two nodes in the neighborhood of all the nodes were approximately equal.

**Figure 8:**
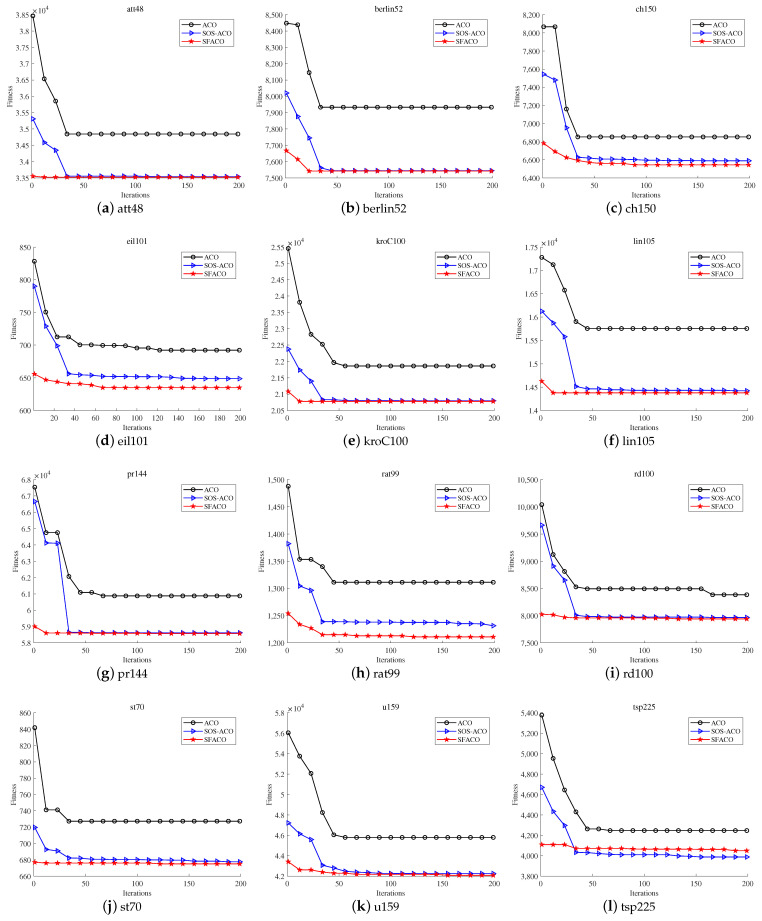
Iterative convergence curves of the ACO, SOS-ACO, and SFACO algorithms.

Figure 8 shows the comparison of the iterative convergence of the ACO, SOS-ACO, and SFACO approaches, from which it can be seen that the SFACO approach outperformed the basic ACO approach in terms of the convergence speed and convergence results in 12 of the datasets (i.e., att48, berlin52, ch150, eil101, kroC100, lin105, pr144, rat99, rd100, st70 u159, and tsp225).

### 4.2. Simulation Application of Sliced Surface Laser Scanning When Based on the SFACO Algorithm

**Figure 9:**
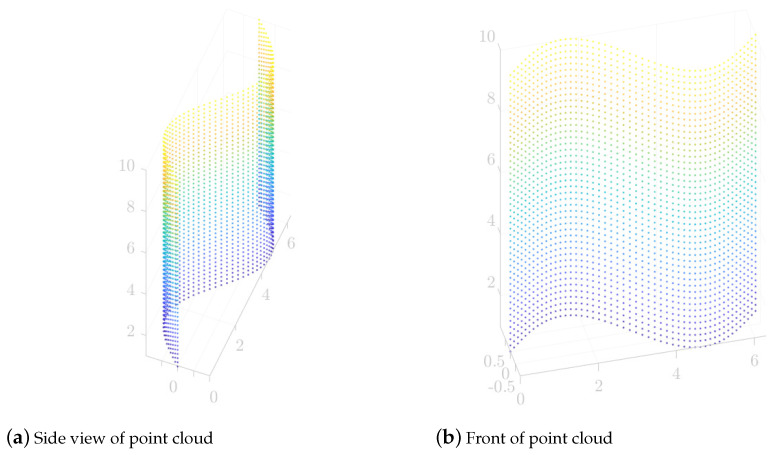
A 3D point cloud data model of the surface.

This part mainly discusses the laser scanning path planning process and results of the SFACO-based segmented surfaces. In this part of the experiment, the 3D point cloud data model of the surface was firstly established as shown in Figure 9, where Figure 9a,b are the side and front display effects of the surface, respectively. The surface point cloud model established in this paper had 2300 points distributed in 3D space, and all of the points were $x\in \left[0,6.2832\right]$, $y\in \left[-0.9995,0.9995\right]$, and $z\in \left[1,10\right]$.

During laser scanning, the laser probe traversed all the points to be measured in a certain order. By optimizing the measurement order of each of the points to be measured, the laser probe movement time can be reduced, effective obstacle avoidance can be performed, and repeated measurements can be avoided. In addition, the laser probe does not need to return to the initial point during the scanning process of the 3D surface. Therefore, in surface measurement path planning, it is necessary to adjust the point laser displacement sensor probe’s attitude to realize the best synergy between the laser probe’s measurement speed and measurement accuracy. The attitude of the probe requires the line laser sensor to emit a beam that can be optimally aligned with the normal direction of the surface being measured. Therefore, in this part of the experiment, the position of the object to be measured in the measurement space $\left(\theta_{x},\theta_{y},\theta_{z},\theta_{w}\right)$ under the optimal measurement attitude of the laser probe was firstly calculated according to Equation (8), which was used in the design scheme of the laser measurement attitude that was proposed in Section 2. In this part of the experiment, the actual examples of the motion parameters of the measuring machine under the optimal measurement attitude were calculated from the surface of Figure 9, which are shown in Table 3. Table 3 shows the coordinates of the first 15 points to be measured on the surface as an example. The left side of the table shows the positions of the first 15 points to be measured on the surface in the coordinate system of the measuring machine, and the right side of the table shows the parameters of the motion of the measuring machine in the optimal measuring attitude $\left(\theta_{x},\theta_{y},\theta_{z},\theta_{w}\right)$ for the points to be measured in the inverse solution.

After calculating the $\left(\theta_{x},\theta_{y},\theta_{z},\theta_{w}\right)$, the nominal distance matrix between the points to be measured with practical significance was constructed according to the position of each point in the measuring machine through Equations (10) and (11), and the constructed nominal distance matrix was input into the proposed SFACO approach to calculate the optimal measurement path of the surface. Table 4 shows the comparison of the ACO, SOS-ACO, and SFACO measurement path planning results. Table 4 mainly shows the average, optimal value, and standard deviation of the path lengths of the ACO, SOS-ACO, and SFACO approaches, which were run independently 10 times each to calculate the path lengths of the surface of Figure 9. It can be seen that the shortest scanning path was obtained from the SFACO planning.

**Figure 10:**
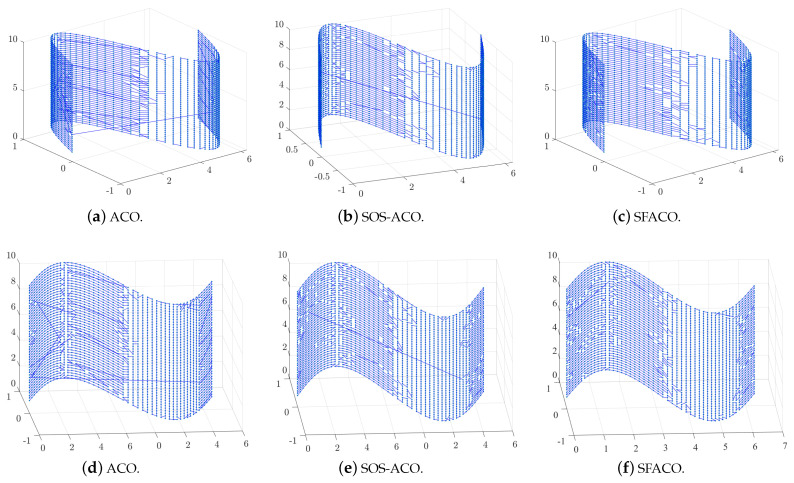
Plots of the ACO, SOS-ACO, and SFACO approaches for the optimal planning measurement paths.

Figure 10 shows the optimal measured path diagram obtained by the ACO approach with SFACO calculation. Figure 10a,d show the optimal path diagram obtained by the ACO approach for path planning calculation. The path length calculated by the ACO approach was 441.4736, and it can be seen from the figure that there were many cluttered routes in the path diagram, which were obtained based on the calculation of the ACO approach. It was observed that the path planning did not reach the optimal state. Figure 10b,e show the optimal path diagram that was obtained by the SOS-ACO approach for the purpose of path planning calculation, and the path length was calculated as 407.1513. It can be seen that the SOS-ACO approach calculated the images more regularly than the ACO approach, but there are still some paths that were messy. Figure 10c,f show the optimal path diagram obtained by the SFACO approach for the purpose of path planning calculation, and the path length calculated was 405.997174156896. Figure 10c,f show the path result, and the scanning path was found to be more regular compared to the results shown in Figure 10a,d and Figure 10c,f. In addition, we can divide the whole surface from left to right into four regions, and it can be seen that they are different. Furthermore, it can be seen that the surface path planning scheme was not the same for the different curvatures, and there was less interspersing, which usually occurred when scanning from left to right. Combined with the optimal measurement posture of the laser probe, surfaces with the same curvature will be scanned centrally and then transferred to other surface areas. Therefore, the scanning paths obtained by the SFACO approach were very consistent with the optimal measurement attitude.

### 4.3. Analysis of the SFACO Algorithm

The SFACO algorithm proposed in this manuscript was proved to be effective by the TSP planning benchmark dataset, and the path planning results were subsequently obtained by applying the SFACO approach to 3D surface laser scanning path planning. However, based on the TSP dataset path planning results, the superiority of the SFACO algorithm proposed in this manuscript was not found to be significant in the case of more nodes. Therefore, subsequent improvements are needed to enhance the performance of the algorithm in large-scale node path planning problems. The population diversity of the algorithm can be further improved by improving the evolutionary strategy, and the global search performance of the algorithm can be improved while the local search capability is strengthened. In addition, the computational steps of the algorithm were reduced to decrease the complexity of the algorithm and increase the computational speed of the algorithm in instances of large-scale node localization in order to improve the practicality of the algorithm in terms of real production.

## 5. Conclusions

This paper introduces the SFACO algorithm, a novel approach to path planning for the 3D laser scanning of intricate surfaces when using a four-coordinate measuring instrument based on point laser displacement sensors. Using a combination of adaptive heuristic factors and a population diversity expression scheme, which was based on prior knowledge and enhances the convergence of the population while ensuring the diversity of the population, a sub-population strategy was created by simulating the human sociology department. The SFACO algorithm utilizes a dynamic domain structure to modify the ant colony paths during the iteration process in order to increase population diversity. At long last, fuzzy logic control has been implemented to modify the pheromone volatilization parameters as the evolution progresses in order to maximize the efficiency of the algorithm for the preceding generation’s experience. The SFACO algorithm proposed in this paper has been tested experimentally, and 13 benchmark test datasets of TSP have been chosen to validate the algorithm. The results of the experiments demonstrate that the SFACO approach outperforms other path planning schemes. Ultimately, the SFACO algorithm was employed to devise the most effective path planning plan for intricate surface 3D laser models.

## Figures and Tables

**Table 1:** The ACO parameters.

Parameters	Value
Population size	m=50
Initial value of the pheromone importance factor	α=1
Initial value of the heuristic function importance factor	β=5
Initial value of the pheromone volatilization factor	$\tau_{0}=0.2$

**Table 2:** Calculation results of the TSP problem.

		GA	ESA	FPSO	IBA	MMAS	ACO	SOS-ACO	SFACO
	avg.	-	-	33,585.70	-	33,576.40	35,834.32	33,539.49	**33,528.40**
att48	best	-	-	33,522.00	-	33,522.00	34,845.64	33,523.71	**33,522.00**
	std	-	-	-	-	-	481.26	15.78	**12.80**
	avg.	7542.00	7542.00	7542.00	7542.00	7596.00	8141.72	7544.37	**7542.00**
berlin52	best	7542.00	7542.00	7542.00	7542.00	7542.00	7933.38	7544.37	**7542.00**
	std	0.00	0.00	0.00	0.00	54.39	94.50	0.00	**0.00**
	avg.	-	-	**6545.40**	-	6552.00	6908.25	6589.38	6568.30
ch150	best	-	-	**6528.00**	-	6528.00	6852.33	6582.31	6544.00
	std	-	-	17.45	-	24.09	61.35	7.73	**13.11**
	avg.	673.80	658.40	636.50	646.40	**636.10**	705.52	648.80	638.50
eil101	best	655.00	650.00	630.00	634.00	629.00	692.07	644.95	**629.00**
	std	12.50	4.40	7.60	-	7.18	9.16	3.38	**2.20**
	avg.	21,510.40	21,170.40	20,812.40	21,050.00	20,812.70	22,115.20	20,798.85	**20,789.30**
kroC100	best	20,861.00	20,749.00	20,749.00	20,749.00	20,749.00	21,864.01	20,780.22	**20749.00**
	std	390.20	188.70	63.59	-	63.90	146.73	20.76	**14.21**
	avg.	-	-	14,454.40	-	14,458.60	16,022.69	14,423.45	**14,404.00**
lin105	best	-	-	14,379.00	-	14,379.00	15,754.01	14,406.12	**14,379.00**
	std	-	-	73.77	-	80.04	174.09	17.35	**26.92**
	avg.	-	-	109,470.50	-	109,646.00	123,569.31	108,326.58	**108,295.40**
pr76	best	-	-	108,159.00	-	108,159.00	121,710.56	108,304.51	**108,159.00**
	std	-	-	1327.40	-	1507.44	1162.73	34.36	**50.85**
	avg.	60,591.40	58,807.30	58,679.30	**58,537.00**	58,560.30	61,315.75	58,615.95	58,602.10
pr144	best	58,599.00	58,574.00	58,537.00	58,537.00	58,537.00	60,877.45	58,602.32	**58,537.00**
	std	2342.80	220.90	142.65	-	23.31	203.68	22.13	**16.72**
	avg.	-	-	1215.20	-	**1214.50**	1333.32	1231.54	1218.70
rat99	best	-	-	1211.00	-	1212.00	1311.34	1223.12	**1211.00**
	std	-	-	**4.21**	-	3.51	11.30	6.32	6.31
	avg.	709.80	682.10	682.30	679.00	682.60	742.58	677.53	**676.00**
st70	best	675.00	675.00	675.00	675.00	675.00	727.32	677.11	**675.00**
	std	5.70	3.90	7.38	-	7.69	8.63	0.63	**0.77**
	avg.	-	-	42,202.50	-	**42,159.50**	46,244.16	42,246.48	42,187.80
u159	best	-	-	42,080.00	-	42,080.00	45,793.52	42,193.08	**42,080.00**
	std	-	-	122.86	-	79.65	294.47	44.64	**55.64**
	avg.	-	-	**3972.10**	-	3971.00	4291.19	3987.72	4026.85
tsp225	best	-	-	**3916.00**	-	3919.00	4246.60	3970.64	3985.00
	std	-	-	56.90	-	55.77	30.42	10.49	**16.69**

**Table 3:** Examples of the surface part coordinates.

Before			After			
x	y	z	$\theta_{x}$	$\theta_{y}$	$\theta_{z}$	$\theta_{w}$
0.000000	0.000000	1	−0.18109	14.99851	1	0.775792
0.128228	0.127877	1	−0.36066	14.99310	1	0.760739
0.256457	0.253655	1	−0.53710	14.98055	1	0.736746
0.384685	0.375267	1	−0.70865	14.95735	1	0.703068
0.512913	0.490718	1	−0.87329	14.91959	1	0.658720
0.641141	0.598111	1	−1.02846	14.86301	1	0.602535
0.769370	0.695683	1	−1.17090	14.78324	1	0.533310
0.897598	0.781831	1	−1.29637	14.67644	1	0.450083
1.025826	0.855143	1	−1.39973	14.54039	1	0.352579
1.154054	0.914413	1	−1.47562	14.37610	1	0.241807
1.282283	0.958668	1	−1.52020	14.18923	1	0.120598
1.410511	0.987182	1	−1.53354	13.99009	1	3.147889
1.538739	0.999486	1	−1.60814	13.90959	1	3.199096
1.666968	0.995379	1	−1.58971	13.71474	1	3.323844
1.795196	0.974928	1	−1.56275	13.53582	1	3.440314

**Table 4:** Comparison of the ACO and SFACO measurement path planning results.

	Avg.	Best	Std.
ACO	444.6934	441.4736	2.5467
SOS-ACO	407.2981	407.1513	0.1304
SFACO	406.3894	405.9972	0.2838

## Data Availability

The data are contained within the article. The dataset cited in this study are available in TSPLIB [29].
